# Genomic convergence and network analysis approach to identify candidate genes in Alzheimer's disease

**DOI:** 10.1186/1471-2164-15-199

**Published:** 2014-03-15

**Authors:** Puneet Talwar, Yumnam Silla, Sandeep Grover, Meenal Gupta, Rachna Agarwal, Suman Kushwaha, Ritushree Kukreti

**Affiliations:** Genomics and Molecular Medicine Unit, Institute of Genomics and Integrative Biology (IGIB), Council of Scientific and Industrial Research (CSIR), Mall Road, Delhi, 110 007 India; Department of Neurochemistry, Institute of Human Behaviour and Allied Sciences, Dilshad Garden, Delhi, India; Department of Neurology, Institute of Human Behaviour and Allied Sciences, Dilshad Garden, Delhi, India

**Keywords:** Gene prioritization, Protein-protein interaction, Clustering, Functional annotation

## Abstract

**Background:**

Alzheimer’s disease (AD) is one of the leading genetically complex and heterogeneous disorder that is influenced by both genetic and environmental factors. The underlying risk factors remain largely unclear for this heterogeneous disorder. In recent years, high throughput methodologies, such as genome-wide linkage analysis (GWL), genome-wide association (GWA) studies, and genome-wide expression profiling (GWE), have led to the identification of several candidate genes associated with AD. However, due to lack of consistency within their findings, an integrative approach is warranted. Here, we have designed a rank based gene prioritization approach involving convergent analysis of multi-dimensional data and protein-protein interaction (PPI) network modelling.

**Results:**

Our approach employs integration of three different AD datasets- GWL,GWA and GWE to identify overlapping candidate genes ranked using a novel cumulative rank score (S_R_) based method followed by prioritization using clusters derived from PPI network. S_R_ for each gene is calculated by addition of rank assigned to individual gene based on either p value or score in three datasets. This analysis yielded 108 plausible AD genes. Network modelling by creating PPI using proteins encoded by these genes and their direct interactors resulted in a layered network of 640 proteins. Clustering of these proteins further helped us in identifying 6 significant clusters with 7 proteins (EGFR, ACTB, CDC2, IRAK1, APOE, ABCA1 and AMPH) forming the central hub nodes. Functional annotation of 108 genes revealed their role in several biological activities such as neurogenesis, regulation of MAP kinase activity, response to calcium ion, endocytosis paralleling the AD specific attributes. Finally, 3 potential biochemical biomarkers were found from the overlap of 108 AD proteins with proteins from CSF and plasma proteome. *EGFR* and *ACTB* were found to be the two most significant AD risk genes.

**Conclusions:**

With the assumption that common genetic signals obtained from different methodological platforms might serve as robust AD risk markers than candidates identified using single dimension approach, here we demonstrated an integrated genomic convergence approach for disease candidate gene prioritization from heterogeneous data sources linked to AD.

**Electronic supplementary material:**

The online version of this article (doi:10.1186/1471-2164-15-199) contains supplementary material, which is available to authorized users.

## Background

Alzheimer’s disease (AD) is a gradually progressive neurodegenerative disease, characterized by cognitive impairment in elderly. Genetics is known to play a major role in its development with studies showing both gene-gene and gene-environment interactions as risk factors [1, 2]. The number of people afflicted with AD is estimated to be more than 24 million worldwide, and the heritability is estimated to be 60-80% [3–5]. Over the last decade, several high throughput experimental approaches involving genome-wide linkage (GWL) scans, genome-wide association (GWA) studies, and genome-wide expression (GWE) profiling, have been extensively utilized to identify the underlying genetic risk factors. Linkage studies were instrumental in the initial identification of four genes (*APP*, *PSEN1*, *PSEN2* and *APOE*) associated with AD [6]. Later, several other loci spanning many genes were discovered in AD using GWL scans. However, linkage studies in sporadic or late onset AD (LOAD) suffers from limitations of low resolution of results, lack of availability of large multigeneration families and inclusion of phenocopies [7].

With the advent of high throughput genotyping platforms in recent years, several GWA studies were carried out using population based case–control designs which resulted in the identification of additional AD risk genes [7, 8]. However, these studies require very large sample size specifically to detect genetic variant with small attributable risk. Additionally, case control studies are prone to issues of population stratification and population admixture. In recent years, a limited number of global gene expression profiling studies have been conducted using post-mortem AD brain tissues [9, 10]. These studies have led to identification of genes related to multiple cellular pathways known to be involved in AD pathogenesis and progression. However, the major drawback of such studies includes limited access to brain samples from AD subjects as well as age matched controls. Further, variable RNA quality due to post-mortem delay and the difficulty in establishing temporal and regional specificity of gene expression changes adds up to the limitations [11]. Although different genetic based approaches have led to the accumulation of massive amounts of data, however, due to differential limitations of each study, limited success has been achieved in identifying common underlying genetic markers related to AD progression and pathogenesis. This warrants designing of novel approaches complementing the existing ones for disease gene discovery.

In recent years, integrative approaches combining multiple data sources have been widely used to identify susceptible genes in complex disorders such as AD [12, 13], epilepsy [14], type 2 diabetes [15, 16], prostate cancer [17], depression [18], schizophrenia [19] and Parkinson’s disease (PD) [20]. Such approaches may help imbibe disease specific biological knowledge that may not be available from one dimensional approaches. Further, network modelling of gene-gene and protein-protein interactions (PPI) provides a relatively new integrative approach to understand complex disease and identify disease-related genes [21, 22]. For instance, candidate genes in complex disorders, such as AD [23–27], obstructive sleep apnea [28], heart failure [29], cancer [30] and cardiorenal syndrome [31], have already been explored extensively using PPI based approach. Thus, a convergent analysis approach involving multi-dimensional datasets combined with network or pathway analysis might serve as a comprehensive approach for disease candidate gene prioritization.

In this study, we aimed to develop a system biology approach based on genomic convergence of genetic data from multiple high-dimensional genome-wide studies and network modelling of protein-protein interactions to prioritize candidate genes linked to AD. We identified 108 common overlapping genes from integrated analysis of three datasets - GWL [8, 32, 33], GWA [34] and GWE [[35, 36]; GSE5281] and ranked them using our ranked based scoring method. We identified direct protein interactors of 108 candidate genes and then created a layered PPI network comprising of 640 nodes based on subcellular localization of proteins. Finally, we performed Markov Cluster algorithm (MCL) based clustering using clusterMaker and functional enrichment analysis using the Database for Annotation, Visualization and Integrated Discovery (DAVID) to identify functional modules and significant Gene Ontology (GO) annotation clusters, respectively [37–39]. Hence, integrating AD linkage, genetic association, and gene expression data followed by network modelling of PPI resulted in a list of evidence-based candidate genes for future experimental validation and related pathways for better understanding of underlying AD patho-physiology. This multi-dimensional evidence-based approach can be applied to other complex disorders having publically available high throughput data.

## Results

**Figure 1:**
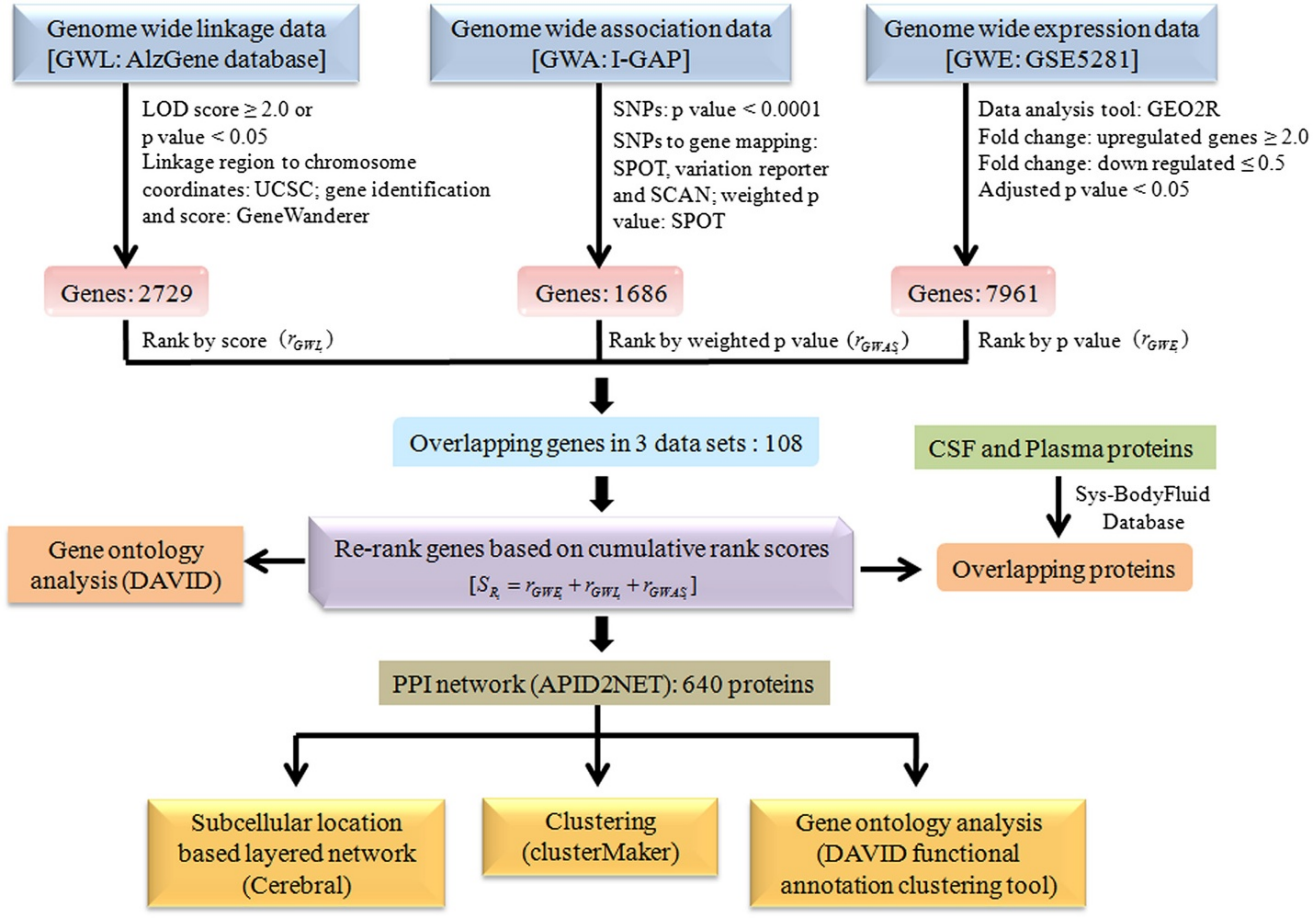
**Flow chart describing the entire work flow.** Integrated data analysis was performed on three genome wide datasets to identify overlapping 108 AD putative candidate genes which were ranked by using cumulative rank based scoring method. These genes were further used to create a PPI and identify overlapping proteins among 108 and proteins from CSF and plasma proteome. PPI was then used to create a layered network based on the subcellular localization information of 640 genes, to identify clusters using MCL algorithm and to retrieve functional annotation using DAVID web tool.

The objective of this study was to identify potential candidate genes involved in AD development and progression by an integrative genomic convergence approach involving rank based scoring method. The datasets, for integrative analysis, were retrieved from AlzGene database (GWL), I-GAP (International Genomics of Alzheimer’s Project) study (GWA) and NCBI Gene Expression Omnibus (GEO) database: GSE5281 (GWE). The common overlapping genes occurring in all the three datasets were identified and ranked by cumulative rank score obtained by addition of gene ranks based on either p values or scores. The final 108 overlapping genes were used for ‘GO analysis’ and to create a layered PPI network comprising 640 nodes and 2214 edges. These identified putative proteins were then used to identify functionally important clusters and common biomarkers among plasma/serum and CSF proteome. The entire work flow is depicted in Figure 1.

### Putative AD linked candidate genes from integrative analysis

For GWL data analysis, genomic linkage regions linked to AD were retrieved from AlzGene database with LOD scores ≥ 2.0 or p value <0.05 (1p31.1-q31.1, 3q12.3-q25.31, 6p21.1-q15, 7pter-q21.11, 8p22-p21.1, 9q21.31-q32, 10p14-q24, 17q24.3-qter, 19p13.3-qter) and used for further analysis. Among these 9 linkage regions, 7 were included from meta-analysis of five independent genome scans carried out by Butler et al. [32], using genome search meta-analysis (GSMA) approach and 2 regions from Hamshree et al. [33] that combined three large samples to give a total of 723 affected relative pairs (ARPs) and analyzed using multipoint, model-free ARP linkage analysis approach. A total of 2976 genes were retrieved using UCSC genome browser [40] from these linkage regions and genes were ranked according to their score obtained from GeneWanderer web server [41].

Further, for the GWA dataset, 19,532 single nucleotide polymorphisms (SNPs) with p value <0.0001 [34] were selected. These SNPs were mapped to their corresponding genes using NCBI Variation Reporter, SCAN (SNP and CNV Annotation) database [42] and SPOT web tool [43]. This led us to the identification of 1,686 genes which were ranked based on weighted p value obtained though genomic information network prioritization and scoring method implemented in SPOT [43]. For replication analysis, we used another GWA dataset from Boada et al. [44] which included genotyped and imputed SNPs (1,098,485) from 7 reported GWA studies comprising ~8082 cases and ~12040 controls for stage I meta-analysis. With this cohort used in stage I analysis with P < 0.001, 1202 SNPs were obtained. When candidate genes identified in the main and replication datasets were compared, we found a concordance of 35.4% (see Additional file 1).

For GWE data analysis, the GSE5281 dataset was selected and analyzed using GEO2R tool accessed from GEO web server [45]. In our study, expression data from six brain regions – entorhinal cortex (EC), hippocampus (HIP), posterior cingulate cortex (PC), middle temporal gyrus (MTG), superior frontal gyrus (SFG) and primary visual cortex (VCX), were used for analysis. The genes with adjusted p value < 0.05 and fold change ≥ 2.0 for upregulated genes and ≤ 0.5 for down regulated genes were selected from each region and then merged. This analysis resulted in 7961 genes which were ranked by their corresponding adjusted p values. For replication analysis, we used another GWE dataset - GSE15222 that comprised expression data from post-mortem brain cortical regions of 176 late-onset AD cases and 188 controls [46]. A concordance of 58.2% was found between GSE5281 and GSE15222 datasets after analysis (see Additional file 1).

**Figure 2:**
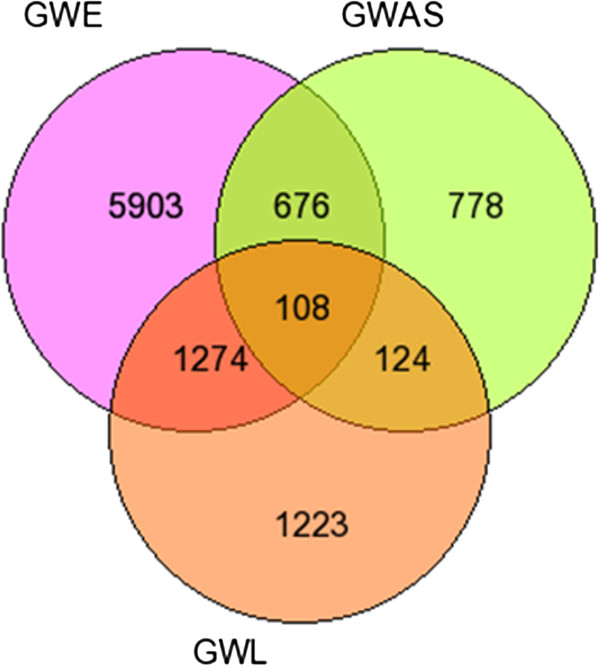
**Venn diagram of putative overlapping AD candidate genes among different genome wide datasets.** The venn diagram represents the genes in the three individual datasets and overlapping 108 putative AD target genes identified by integrated analysis of the three datasets.

The intersection of all the three datasets resulted in the final set of 108 putative candidate genes (Figure 2) and their individual ranks were added to get  score. Based on this rank score the genes were re-ranked with gene having the lower cumulative rank score getting the higher rank. The top 10 genes are listed in Table 1 and the list of 108 genes is provided in Additional file 2.

**Table 1:** **Top 10 genes from the list of 108 target genes found in the overlap of three data sets**

S.No.	Gene Symbol	Gene Name	Rank_	Rank_	Rank_	Cumulative rank score	Final rank	HGNC ID	Location
			GWL	GWA	GWE				
1	*RPN1*	Ribophorin I	232	61	324	617	1	HGNC:10381	3q21.3
2	*RGS4*	Regulator of G-protein signaling 4	240	300	97	637	2	HGNC:10000	1q23.3
3	*HIP1*	Huntingtin interacting protein 1	548	29	303	880	3	HGNC:4913	7q11.23
4	*PTK2B*	Protein tyrosine kinase 2 beta	148	14	879	1041	4	HGNC:9612	8p21.1
5	*ICA1*	Islet cell autoantigen 1, 69 kDa	323	453	268	1044	5	HGNC:5343	7p22
6	*AMPH*	Amphiphysin	540	539	194	1273	6	HGNC:471	7p14-p13
7	*ATP5H*	ATP synthase, H + transporting, mitochondrial Fo complex, subunit d	817	279	192	1288	7	HGNC:845	17q25
8	*EGFR*	Epidermal growth factor receptor	24	434	909	1367	8	HGNC:3236	7p12
9	*ABCA1*	ATP-binding cassette, sub-family A (ABC1), member 1	47	217	1138	1402	9	HGNC:29	9q31
10	*ACTB*	Actin, beta	49	1348	10	1407	10	HGNC:132	7p22

As all the six brain regions are found to be associated with AD pathology with different degree of involvement depending upon disease severity, we analysed expression profile data of each region separately and obtained candidate genes specific in each brain region. We identified 25, 16, 40, 38, 27 and 1 candidate genes specific in EC, HIP, PC, MTG, SFG and VCX brain regions, respectively, from overlap with GWA and GWL repertoires (see Additional files 3 and 4).

### Protein-protein interaction network, layering and network analysis

**Figure 3:**
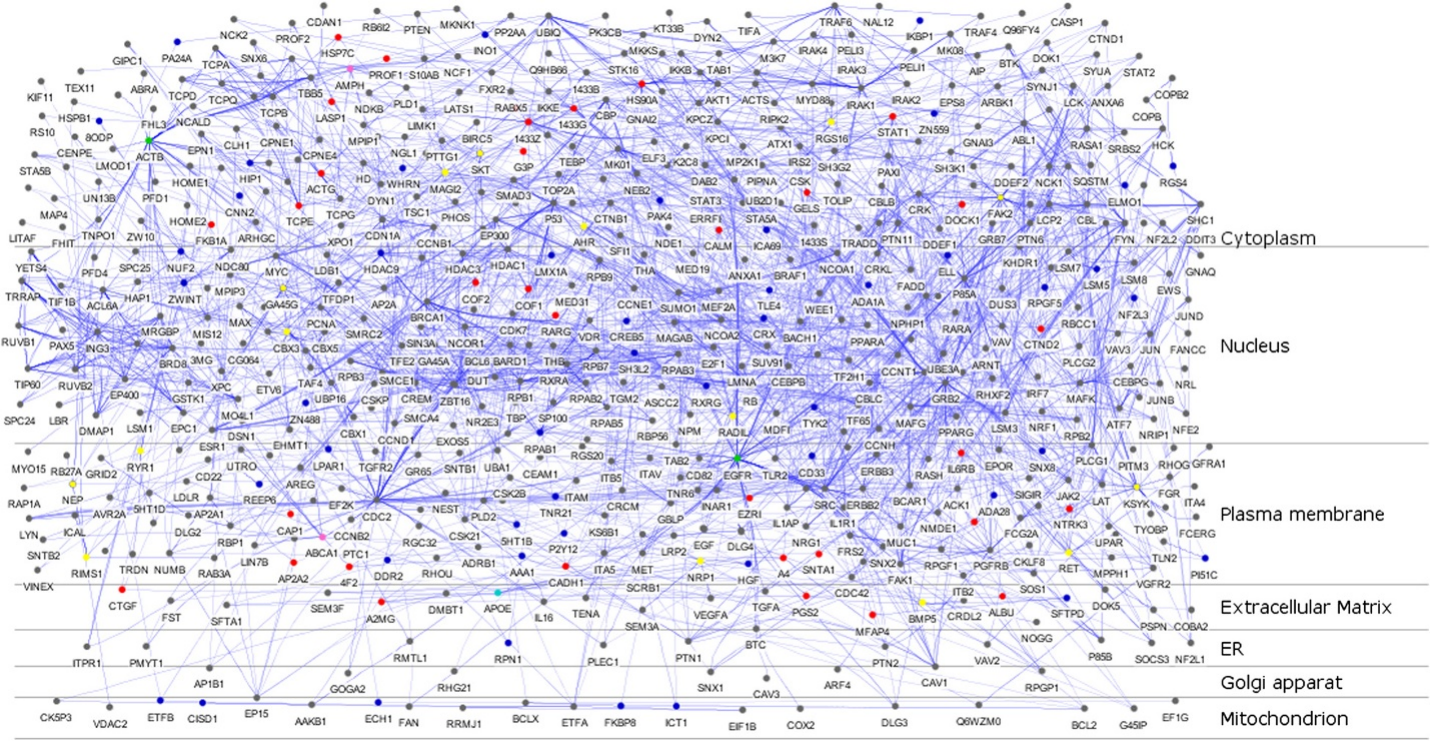
**Layered Protein-Protein Interaction network (PPI) of 108 proteins.** A layered network based on the subcellular localization of 640 proteins in the PPI was created. The nodes representing functionally important genes were highlighted in the layered network using colour codes - green (genes forming hub nodes in clusters (7), occurring in top 15 of ranked genes (108) and also present in putative biomarker dataset (38)); cyan (genes forming hub nodes in clusters, occurring in 108 AD genes and also present in putative biomarker dataset); yellow (genes occurring both in cluster hub and in 108 ranked genes); pink (genes forming hub nodes in clusters and occurring in top 15 of ranked genes); blue (remaining 59 from 108 list); red (38 biomarkers from AD, CSF and plasma overlap); grey (remaining proteins from 640 candidates).

**Figure 4:**
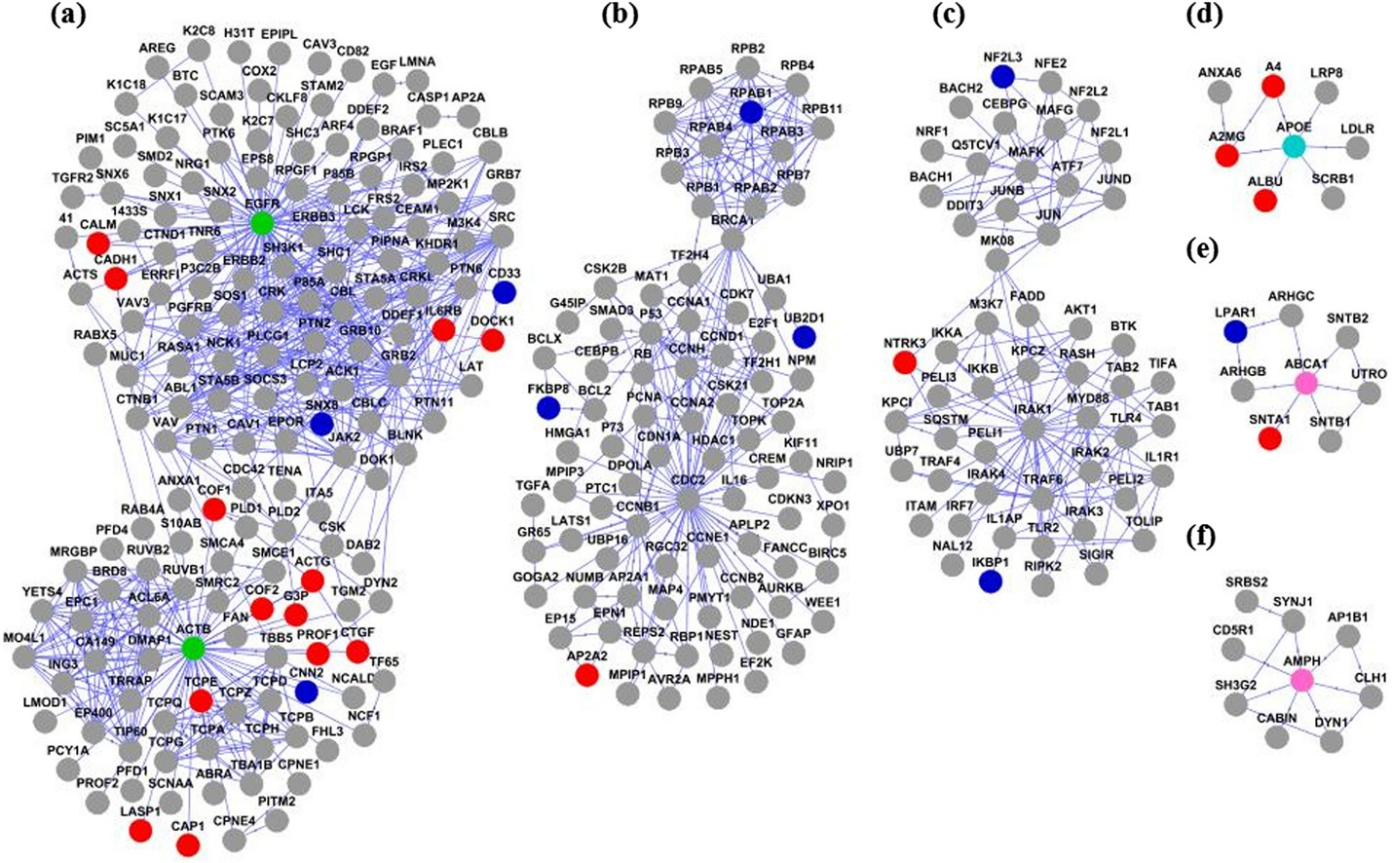
**Important clusters obtained from clustering of 640 proteins using MCL algorithm in clusterMaker. (a-f)** Biologically significant gene clusters were identified from PPI using MCL algorithm. The nodes representing functionally important genes were coloured in the pattern described for the layered network.

Identification of proteins that interact directly with proteins encoded by identified 108 target genes might help elucidate the molecular mechanism underlying AD patho-physiology. Thus, in the present study, we created a PPI network from the 108 candidate genes using APID2NET plugin in Cytoscape [47, 48] comprising 640 nodes and 2214 edges. Then, a layered network based on the sub-cellular localization information of 640 proteins using “Cerebral” plugin [49] in Cytoscape was obtained from the PPI network. The layered network is depicted in Figure 3. Further, another cytoscape plugin “clusterMaker” [37] was used on the PPI to create clusters using MCL clustering algorithm [50]. This resulted in the identification of 6 important clusters with 7 proteins (EGFR, ACTB, CDC2, IRAK1, APOE, ABCA1 and AMPH) forming the central hub nodes (Figure 4a-f). All 63 clusters obtained from MCL clustering are provided in Additional file 5.

### Functional annotation analysis by GO terms

**Figure 5:**
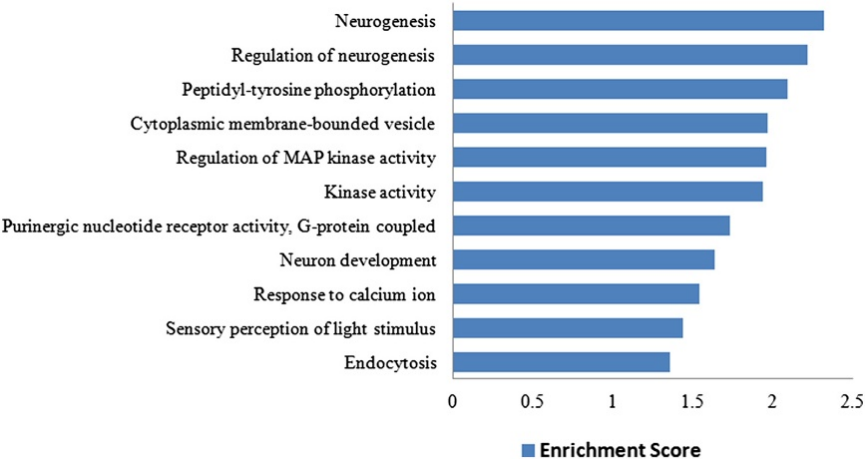
**Clustering of GO terms: significantly over represented top 11 functionally annotated clusters from biological process, cellular component and molecular function of 108 proteins.**

We performed functional GO enrichment analysis of the 108 AD candidate genes, using functional annotation clustering tool implemented in DAVID [38, 39], to identify association of candidate genes with different ‘GO terms’. The significantly over represented ‘GO terms’, identified neurogenesis (p = 0.0032) as the top cluster, followed by regulation of neurogenesis (p = 0.0062). The other significantly over represented biological processes included peptidyl tyrosine phosphorylation (p = 0.0041), cytoplasmic membrane-bounded vesicles (p = 0.006), regulation of MAP kinase activity (p = 0.0005), kinase activity (p = 0.0081), purinergic nucleotide receptor activity, G-protein coupled (p = 0.0153), neuron development (p = 0.0098), response to calcium ion (p = 0.0067), sensory perception of light stimulus (p = 0.0041), endocytosis (p = 0.0192) (Figure 5). This analysis was also repeated for 640 candidate genes (Additional file 6).

### AD putative biochemical biomarkers

**Figure 6:**
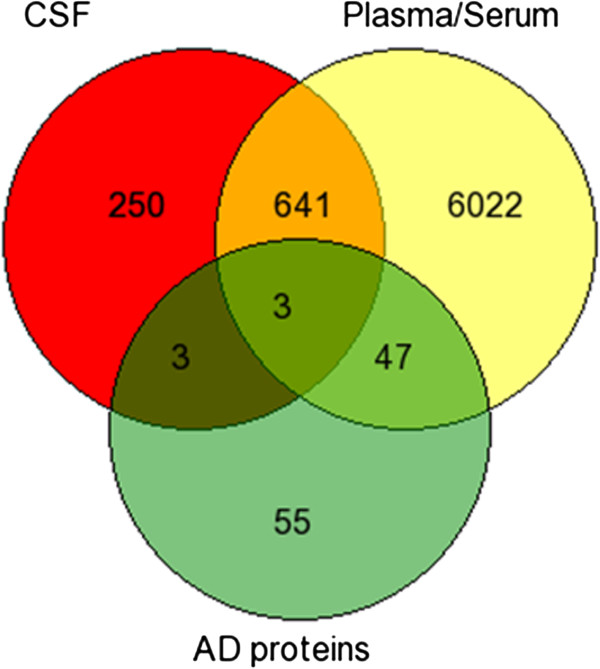
**Putative AD specific biomarkers.** The venn diagram depicts overlap among putative 108 AD proteins, proteins from CSF and plasma proteome.

In this study, we also looked for the identification of cerebrospinal fluid (CSF) and plasma based AD specific biomarker and found 3 common proteins (APOE, EGFR, ACTB) among 108 AD proteins and proteins from CSF and plasma proteome (Figure 6) and 38 common proteins among 640 putative AD proteins and proteins from CSF and plasma proteome (Additional file 7), which might serve as potential biochemical biomarkers for early detection of AD cases in future.

### Validation of Rank based approach by using PD datasets

For validation of our rank based gene prioritization approach, we selected PD, another common complex neurodegenerative disorder that involves the deposition of α-synuclein as intracellular Lewy bodies leading to progressive degeneration of dopaminergic neurons within multiple brain regions. It clinically manifests as both a movement disorder, characterized by tremor, rigidity, bradykinesia and postural instability and a distinct form of cognitive impairment, characterized by visuospatial impairment and fluctuations in mental state [51, 52]. We applied our rank based method to identify overlapping genes in three PD datasets – GWL, GWA, and GWE. We retrieved GWL dataset from PDgene database (http://www.pdgene.org/) [53]. It included genetic loci showing evidence for linkage in the meta-analysis of five GWL scans comprising 862 families with 1384 affected subjects using the GSMA method by Rosenberger et al. [54].

For GWA dataset, we retrieved SNPs with pre-computed p values from a NCBI dbGaP database with study accession: phs000089.v3.p2 (http://www.ncbi.nlm.nih.gov/gap) [55]. This dataset comprises PD cases drawn from population of North American Caucasians, and neurologically normal controls from the population which are banked in the National Institute of Neurological Disorders and Stroke (NINDS Repository) collection for a stage I genome wide analysis. Initially, genome-wide, SNP genotyping of these samples was carried out in 267 PD subjects and 270 controls, and later extended to include genotyping in 939 PD cases and 802 controls. This collection was included in the first stage study by Fung et al. [56], and the expanded study by Simon-Sanchez et al. [57, 58]. A total of 7,943 SNPs (stage I) were selected for further analysis, with p value < 0.01, from raw data comprising total of 453,217 SNPs.

For GWE dataset, we selected the gene expression data from NCBI GEO database (GSE20295) (http://www.ncbi.nlm.nih.gov/geo) [59] for analysis. It contained gene expression profiling data in post-mortem tissue of three brain regions (the substantia nigra, putamen, and Brodmann’s area 9) from matched groups of 15 neuropathologically confirmed PD and 15 controls with no history of major brain illness.

**Figure 7:**
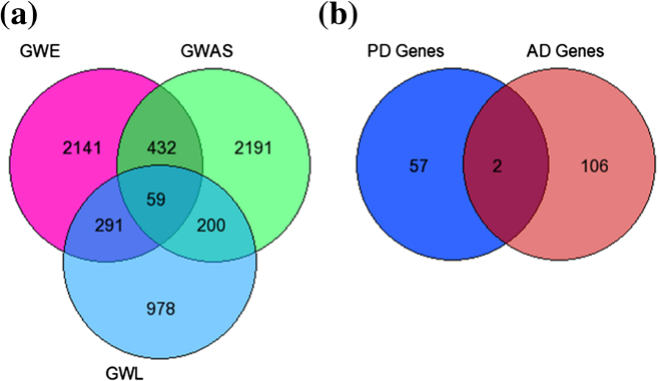
**Prioritized putative PD candidate genes and overlap with prioritized AD candidate genes. (a)** The venn diagram represents the genes in the three individual datasets and overlapping 59 putative PD target genes identified by integrated analysis of the three datasets. **(b)** The venn diagram represents the overlapping genes among AD and PD putative target genes.

The analysis of three PD datasets using rank based scoring method led us to the identification of 59 putative target genes from the overlap of 1528 genes from GWL, 2882 genes from GWA and 2923 genes from GWE which could have significant association with PD development and progression (Figure 7a). The entire list of 59 genes is provided in Additional file 8. The comparison of 108 AD and 59 PD putative candidate genes resulted in only 2 common genes (*ABCA1* and *LPAR1*) between the two groups (Figure 7b).

## Discussion

AD is a complex polygenic disorder with lack of understanding of natural course of the disorder and absence of reliable biomarkers that can predict disease onset and progression. Although, genome-wide studies, such as genetic linkage, association and expression, have allowed unbiased identification of candidate genes and pathways associated with AD development and progression, susceptibility loci or genes for AD with clinical significance have not yet been reported. This can be attributed to certain limitations associated with these methods. For instance, linkage studies require large, multi-generational pedigrees within which both affected and unaffected individuals are required for testing and even in such cases, this approach yields only regions of linkage and not the causative gene [60–62].This suggests that only a fraction of the genes, significant in these analyses, are causal genes. On the other hand, GWA studies often lack statistical power to detect SNPs with small effect size and therefore cannot detect all causal genes [63]. Further, in case of gene expression studies, identified genes are expected to contain a mix of causal and the differentially expressed genes because of the ripple effect of the causal genes [64]. The huge amount of AD specific genetic data accumulated in the past decade also indicates involvement of multiple pathways wherein each gene confers only a modest risk. Therefore, integration of datasets from multiple disciplines may lead to identification of candidate genes from different pathways and may provide an opportunity to uncover the biological functions and molecular mechanisms underlying AD through PPI network and GO analysis.

In this work, we presented a framework for integrated analysis of multi-dimensional datasets by using a rank based scoring method. First, we retrieved and analysed data from three datasets –GWL, GWA and GWE, based on the assumption that genes identified by all the three experimental technique might be significantly involved in AD pathology. Then, we used a ranked based method in which overlapping genes were first identified in all the three datasets and then each gene was assigned cumulative rank score (S_R_), based on addition of corresponding rank in individual datasets. The genes in each datasets were ranked based on either p values or scores. Finally, the genes were ranked based on their S_R_ with gene having lower S_R_ getting the higher rank. This analysis led us to the identification of 108 ranked genes from the overlap of 2729 genes from GWL, 1686 genes from GWA and 7961 genes from GWE which might serve as putative target genes having significant association with AD development and progression.

A majority of top ranked putative candidate genes have been found to be significantly associated with molecular mechanism and pathways related to AD development and progression and may serve as critical candidates for predicting AD risk. For instance, first ranked *RPN1* gene encodes for a type I integral membrane protein, ribophorin that have been shown to directly interact with opioid receptors (OR). Overexpression of RPN1 is reported to enhance cell surface expression of δOR and μOR but not that of κOR [65]. Significant reductions in μOR binding are observed in the subiculum and HIP regions of brain from AD cases as compared to controls. Further, binding of δOR is also found to be decreased in the amygdala and putamen of AD brains [66]. In addition, δOR have been associated with increased processing of amyloid beta (Aβ) precursor protein (APP) by BACE1 and γ-secretase, but not that of Notch, N-cadherin or APLP1. Moreover, knockdown or blocking of δOR in AD mouse model decreases secretase activities and abolishes Aβ pathology and Aβ-dependent behavioral abnormalities [67]. Second ranked gene, *RGS4,* encodes for regulator of G protein signalling 4 protein, is reported to be involved in neuronal calcium dependent signaling, a cellular process related to both AD and aging [68]. In parietal cortex of AD subjects, 53% and 40% lower levels of RGS4 and Gq/11 proteins is found as compared to age-matched controls. Further, it was proposed that alteration of dynamic equilibrium between the cytosolic and membrane levels of RGS4 and Gq/11 may lead to the regional differences in the coupling of muscarinic M1 receptors in AD which in turn may lead to variable response to currently available cholingeric treatment strategies [69]. *HIP1* gene encodes for Huntingtin interacting protein 1 (HIP1) that is predominantly expressed in brain and is proposed as a novel brain tumor marker that interacts with EGFR [70]. In a published genome-wide study of aging, rs17149227 (p value < 10^-5^) close to *HIP1* gene, is found to be associated for time to death from meta-analysis of 9 cohorts [71]. Mills et al. (2005) proposed that transcriptional deregulation of HIP1 may play a significant role in the pathogenesis of neurodegenerative diseases [72].

A recently found strong LOAD candidate is *PTK2B/CAKB/FAK2/PYK2* gene that encodes for a cytoplasmic protein tyrosine kinase, which is highly expressed in the CNS, particularly in HIP [73]. Aβ fibrils has been shown to induce THP-1 cells resulting in the stimulation of PYK2 tyrosine phosphorylation as a consequence of Lyn and Syk activation, intracellular calcium release, and PKC stimulation [74]. Activation of CAKb/Pyk2 is required for inducing long-term potentiation (LTP) in CA1 HIP neurons which may depend upon downstream activation of Src to upregulate N-methyl-D-aspartate-type (NMDA) glutamate receptors [75–77]. Further, in the case of AD, the immunoreactivity for c-Jun is found to be elevated in diseased brain [78, 79] and interestingly, PYK2 represents a stress sensitive mediator of c-Jun N-terminal kinase (JNK) signaling pathways.

*ICA1* encodes for 69 kDa islet cell autoantigen, a BAR (Bin/amphiphysin/Rvs)-domain-containing protein with highest expression levels in brain, pancreas, and stomach mucosa [80]. It is identified as the major binding partner of protein PICK1 (protein interacts with C-kinase 1). ICA1 regulates AMPA receptor trafficking, an important mechanism underlying synaptic plasticity, by forming heteromeric ICA69-PICK1 complexes and preventing formation of PICK1- PICK1 homomeric complexes [81]. Spitzenberger et al. demonstrated that mutation of *ICA69* homologue gene ric-19 in C. elegans leads to impairment of acetylcholine release at neuromuscular junctions suggesting role of ICA69 in neuroendocrine secretion [82]. *AMPH1* gene encodes for protein amphiphysin I, an important regulator for synaptic vesicle endocytosis (SVE) when massive amounts of Ca^2+^ flow into presynaptic terminals, a phenomenon observed in AD [83]. In *AMPH1* knockout mice, decreased synaptic vesicle recycling efficiency and cognitive deficits has been observed [84]. In a recent study, AMPH1 level is found to be reduced in AD brain regions known to accumulate aggregates of hyperphosphorylated tau proteins [85]. Further, stimulated neurons are also shown to abnormally accumulate amphiphysin, at the membrane during Aβ treatment [86].

Interestingly, *ATP5H/KCTD2* locus is reported as the major candidate gene associated with AD pathogenesis in the study by Boada et al. [44] that is used in this study as the replication dataset. *ATP5H* gene encodes for mitochondrial ATP synthase that plays an important function in mitochondrial energy production and neuronal hyperpolarization during cellular stress conditions, such as hypoxia or glucose deprivation [44]. *EGFR* gene encodes for epidermal growth factor receptor protein, a cell surface protein that binds to epidermal growth factor. It has been put forward as a preferred target for treating amyloid beta induced memory loss in a recent study by Wang L et al. [87]. Interestingly, it has come up as one of the most significant candidate in our study occurring in top 10 ranked genes among 108 candidates, as central hub node in cluster and in the overlap of AD protein and proteins from plasma and CSF proteome. Increased expression of *EGFR* is observed in fibroblasts deficient in PS/gamma-secretase activity or APP expression [88]. Further, studies also indicate role of PS1 in trafficking and turnover of EGFR as well as perturbed endosomal-lysosomal trafficking in cell cycle control and Alzheimer disease and suggest potential pathogenic effects of elevated EGFR [89]. In a recent study, altered EGFR transcript levels are reported among APOE4 (high risk) when compared to APOE3 (low risk) genotype groups [90].

A major candidate gene for LOAD due to its role in cholesterol transport and metabolism is *ABCA1* gene that encodes for ATP-binding cassette transporter A1, a membrane-associated protein. Increased expression of *ABCA1* is highly correlated with severity of dementia in AD HIP [91]. Further, ABCA1 has been shown in mouse models of AD to enable the clearance of Aβ from the brain, through its role in the apolipoprotein (APOE) lipidation in the CNS [92–95]. In APP transgenic mice, ABCA1 deficiency increased Aβ deposition in the brain paralleled by decreased levels of ApoE [96]. In addition, ABCA1 is also found to be up-regulated in primary mouse cortical neurons and cultured astrocytes in response to oligomeric Aβ42 [97, 98]. Recent studies pointed out that ABCA1 mediates the beneficial effects of the liver X receptor (LXR) agonist GW3965 on object recognition memory and amyloid burden in APP/PS1mice [99, 100]. Based on strong evidence the LXR-ABCA1-APOE regulatory axis is now considered a promising therapeutic target in AD [101]. However, a meta-analysis report of 13 studies involving a total of 12,248 subjects failed to find association of common SNPs in *ABCA1* with AD risk [102]. In contrast, Lupton et al. in a very recent study sequenced all *ABCA1* coding regions in 311 LOAD cases and 360 control individuals of Greek ethnicity and observed significantly higher proportion of rare non-synonymous variants in control individuals compared to AD cases. These findings suggest that high throughput sequencing may identify rare variants that are left undetected by GWAS [92]. *ACTB* gene encodes for protein β-actin. It is found to have the worst candidate with reliable expression among a set of suitable endogenous reference genes (ERG) in human post-mortem brain when used for the expression analysis of potential candidate genes associated with AD [103]. *ACTB* was found to be upregulated by 10.2 folds in AD cerebral cortex compared with age-matched control brain [104]. Further, immunoprecipitation of proteins from AD and control brain showed oxidative modification of β-actin in the AD brain [105]. In addition, β-Secretase-cleaved APP is shown to accumulate at actin inclusions in neurons induced by stress or Aβ [106]. Several recent studies also indicate that abnormalities of actin cytoskeleton may play a critical role in AD pathology by mediating synaptic degeneration [107, 108].

We aimed to identify direct protein interactors of proteins encoded by identified 108 candidate genes by PPI network modelling with an assumption that they might provide important biological information related to molecular mechanisms underlying AD development and progression. PPI network was obtained by using APID2NET plugin in Cytoscape and included 640 protein nodes and 2214 edges. It was converted to a layered network based on subcellular localization information. We observed that majority of the proteins were localized in cytoplasm followed by nucleus. Further, we applied MCL clustering algorithm to identify functional modules with proteins forming hub nodes (EGFR, ACTB, CDC2, IRAK2, APOE, ABCA1 and AMPH) which might serve as important candidates related to AD [50]. For instance, CDC2 [109, 110], IRAK2 [111] have been reported in recently published studies with suggestive role in AD pathogenesis. GO analysis was also carried out using 108 genes to identify biological processes, molecular functions and cellular components. Top 11 annotation clusters with enrichment score > 1.3 included genes involved in diverse biological processes related to AD such as neurogenesis (*DFNB31, PTK2B, RET, DLL3, APOE, CRX, ACTB, NRP1, LMX1A, PIP5K1C, ZNF488*), regulation of neurogenesis (*DLL3, APOE, CRX, NRP1, LMX1A, ZNF488*), peptidyl tyrosine phosphorylation (*TYK2, PTK2B, DDR2, SYK*), cytoplasmic membrane-bounded vesicles (*PLA2G4A, ABCA1, HIP1, AMPH, HGF, SFTPD, EGFR, ICA69, ATP8B3, RPN1*), regulation of MAP kinase activity (*PTK2B, LPAR1, APOE, RGS4, HGF, EGFR, SYK*), kinase activity (*PTK2B, TYK2, NME8, DDR2, NRP1, PIP5K1C, EGFR, RET, PAK4, IPMK, POLR2E, ADK, SYK*), purinergic nucleotide receptor activity, G-protein coupled (*SUCNR1, P2RY12, P2RY14*), neuron development (*DFNB31, PTK2B, RET, ACTB, NRP1, LMX1A, PIP5K1C, EGFR*), response to calcium ion (*PLA2G4A, PTK2B, ACTB, EGFR*), sensory perception of light stimulus (*DFNB31, RGS16, PCDH15, CRX, RIMS1, ELOVL4, OPN5*), endocytosis (*ABCA1, APOE, HIP1, AMPH, ELMO1, SFTPD*).

In addition, potential CSF and plasma/serum based biomarkers were identified from the overlap of 108 and 640 AD proteins separately with proteins from CSF and plasma proteome. This resulted in the identification of 3 proteins and 38 proteins as potential biochemical biomarkers for AD among 108 and 640 identified protein datasets, respectively. Among these proteins, the CSF or plasma level of, APOE [112–120], EGFR [121] proteins have been reported to be altered in previous AD studies.

For validation of our approach, we have applied our rank based scoring method to identify PD candidate genes using three (GWL, GWA and GWE) datasets and then we compared PD candidate genes with those identified in analysis of AD datasets to check the robustness of our approach. We failed to find significant overlap in genes between AD and PD dataset in our study, which is further substantiated by a recent meta-analysis carried out by Moskvina et al. that combined the AD and PD GWA studies and failed to identify any significant evidence to support a common genetic risk between AD and PD [122]. Further, the author failed to find loci that associate with increased risk of causing both PD and AD. In addition, it is proposed that the pathological overlap among AD and PD proteins may occur at a later stage during disease progression suggesting interaction of genes from downstream cascade with susceptibility genes that increase the risk of each disease [122]. Few studies investigated simultaneous co-occurrence of AD and PD in families but yielded inconsistent results. In general, studies have reported either no risk of AD in the relatives of subjects with PD or an increased risk of AD in younger subjects with PD or those with cognitive impairment [123–125].

The recent association of several genes identified in our study to AD provides an immediate support of our work and prioritization of such candidates clearly indicates the efficiency and importance of our method. Our approach provides a list of AD candidate genes that are promising for further analysis by exploration of biological functions. The other most common candidate gene prioritization approaches use single-dimentional data-source and are based on direct PPI of the genes that are being studied. However, currently only ~10% of all human PPI have been described which is a major drawback of these approaches [126]. Here, we have tried to address these issues by using multi-dimensional data and exploiting the clustering of PPI network for identification of functional modules. Still, the limitations of our study include constraints in the gene annotation in the selected linkage regions and the availability of raw genome-wide data. Owing to these limitations, it is possible that a few putative candidate genes may have been missed out in this study during the screening process. Further, extensive experimental validation of candidate genes from our analysis is warranted in future.

## Conclusion

To achieve better identification of complex disease associated genes, it is imperative to use integrative approach with disease specific methodologies. We performed integrated analysis of three different datasets – GWL,GWA and GWE and developed a rank based scoring method which resulted in the identification of 108 putative AD candidate genes. Further, network analysis led to a PPI with 640 nodes and clustering of this network resulted in 6 significant clusters with 7 genes forming central hub nodes. Finally, 3 biochemical biomarkers were also identified from the overlapping genes between 108 AD proteins and proteins in CSF-plasma proteome. *EGFR* and *ACTB* were found to be the two most significant AD risk genes ranked 8 and 10 among 108 genes respectively, present as central hub nodes in respective clusters and also as potential biochemical biomarker. We believe that our findings would provide a wealth of information for future experimental and clinical validation in AD pathogenesis and therapeutics.

## Methods

### Genetic linkage data retrieval and processing

We used linkage regions from AlzGene database which were based on the results of meta-analyses [32] and combined analysis [33] of previously published genome-wide linkage (GWL) data. In our study, linkage regions with LOD scores ≥ 2.0 or p value <0.05 linked to AD were selected for further analysis. The chromosomal coordinates for each linkage region were retrieved using UCSC genome browser. These were then used to extract genes from GeneWanderer web server [41] which provides a method for prioritization of candidate genes by using four different ranking strategies (random walk, diffusion kernel, shortest path and direct interaction) on a PPI network. We used random walk since it has been showed to outperform the others [41, 127, 128].

### Genome wide association data retrieval and processing

We used SNPs with pre-computed p values from a recently published GWA study performed under the International Genomics of Alzheimer’s Project (I-GAP) banner [34]. The data are available for download from the following link: http://www.pasteur-lille.fr/en/recherche/u744/Igap_stage1.zip. The study performed meta-analysis on genotyped and imputed data (7,055,881 SNPs) from 4 previously published GWAS [ADGC, CHARGE, EADI, GERD consortium datasets] comprising 17,008 cases and 37,154 controls (stage 1). A total of 19,532 SNPs were found to be associated with AD risk and having p value < 1 × 10^-3^ after stage 1 meta-analysis. For replication analysis, we have used another GWA dataset from Boada et al. [44] that included genotyped and imputed SNPs (1,098,485) from 7 reported GWAS (Antúnez et al. [129], TGEN [130], ADNI [131], genADA [132], NIA [133], Pfizer [134], GERAD [135]) comprising ~8082 cases and ~12040 controls for stage I meta-analysis. With this cohort used in stage I analysis with P < 0.001, 1202 SNPs were obtained. These data are available as Supplementary Table S4 in the study by Baoda et al. [44].

The SNPs were mapped to genes using NCBI variation reporter tool, SCAN database [42] and SPOT tool [43]. SNPs, which remained unmapped, were excluded from further analysis. SPOT tool implements the Genomic Information Network prioritization method and provides a prioritization score that represents an order of magnitude change in p value from a test for association. SPOT score takes into account SNPs functional properties (including nonsense, frameshift, missense and 5’ and 3’-UTR designations), impact of an amino acid substitution on the properties of the protein product from PolyPhen server [136, 137], evolutionary conserved regions from ECRbase [138], all possible LD proxies - SNPs with r^2^ over a predefined threshold in a specific HapMap sample [139].

### Gene expression data retrieval and processing

We retrieved the gene expression data from NCBI GEO (GSE5281) database (http://www.ncbi.nlm.nih.gov/geo) [38]. It contained expression data from six functionally and anatomically distinct regions in human brains, including EC, HIP, MTG, PC, SFG and VCX. The data included 161 samples and each brain region contains AD cases versus normal controls. GEO2R web application, available at http://www.ncbi.nlm.nih.gov/geo/geo2r/, was used for R-based analysis of GEO data [38]. The numbers of samples in each region of control/affected cases were 13/10 of EC, 13/10 of HIP, 12/16 of MTG, 13/9 of PC, 11/23 of SFG and 12/19 of VCX. In our study data from all 6 regions were analysed separately and then merged. For replication analysis, we used another GWE dataset - GSE15222 that comprised expression data from post-mortem brain cortical regions of 176 late-onset AD cases and 188 controls. On the GEO2R web interface, after the GSE5281 series were specified, a table populated with sample characteristics appears. The AD and control sample groups were designated to compare for each brain region separately. Default analysis setting with Benjamini & Hochberg (False discovery rate) for p value adjustments was used.

Probe sets that were not associated with known genes were removed from further analysis. If multiple probe sets represented the same gene and they showed same direction of expression, the probe set with the highest variance was used. If the direction of expression for multiple probe set was different then they were excluded from further analysis. The genes with adjusted p value < 0.05 and fold change ≥ 2.0 for upregulated genes and ≤ 0.5 for downregulated genes were selected. The genes from the six brain regions were merged and duplicates were removed.

### Filtering and scoring of genes from data sets

The genes in all the three datasets were assigned HGNC (HUGO Gene Nomenclature Committee) ids separately [140]. The pseudogene, hypothetical, loci, non-coding RNA, non-protein coding genes, non-functional proteins, open reading frames (orf), chromosome X (Xp; Xq), withdrawn entries, antisense RNA, microRNA, uncharacterized genes were excluded from each data set for further analysis. The genes were ranked in GWL and GWA datasets by score and weighted p value obtained through GeneWanderer [41] and SPOT web servers [43], respectively. The genes in GWE dataset were ranked by adjusted or unadjusted p value obtained after analysis with GEO2R web tool. The genes, with higher weight or lower p value, were assigned higher ranks. The genes, appearing in all the three dataset, were identified and cumulative rank score for each gene was calculated using the following equation -


where,


Based on their rank score the genes were re-ranked with one having the lower cumulative rank score getting the higher rank.

### Protein-protein Interaction network, layering and network analysis

To identify the direct interacting partners of the putative genes identified in this study from integrative analysis of three different data types, we built a PPI network using plugin APID2NET in Cytoscape version 2.8.1 [48] as described by Silla et al. [141]. Briefly, the APID2NET (APID) server creates PPI network of user-provided proteins using literature-curated protein interaction information from various databases such as BIND, BioGrid, DIP, HPRD, IncAct and MINT. UniProt ids of the 108 putative AD target genes were retrieved using uniprot id mapping tool (http://www.uniprot.org) [142] and provided as input ids in APID server to build the interaction network. For creating a PPI network, we first considered only those interactions supported by at least two experimental validations in order to minimize false-positive interactions. However, for proteins lacking interacting partners validated by two experiments, the interacting partners with one experimental validation were considered resulting in another PPI network. Three Cytoscape tools viz Advance Network Analyzer [143], Cerebral [52] and clusterMaker [37] were then applied for modelling PPI network. The Advanced Network Merge was used to model a final PPI network by taking union of both the PPI networks and for removal of duplicated edges and self loops. Isolated nodes were also manually removed from the final PPI network. Protein sub-cellular localization information for 635 proteins were retrieved from uniprot database [142] and for remaining 56 genes from human protein atlas [144] which were imported as node attributes in cytoscape. Then Cytoscape plugin “Cerebral” v.2.8.2 was applied to the final network to layout all nodes according to their sub-cellular localization such as plasma membrane, cytoplasm, nucleus, golgi apparatus, extracellular matrix, endoplasmic reticulum (ER), lysosome and mitochondria. Further, Markov Cluster algorithm (MCL) [50, 145] which was implemented in the “clusterMaker” v.1.9 plugin [37] in cytoscape was used on the PPI to create clusters with the hub nodes. The MCL algorithm has been used specifically to cluster simple graphs and weighted graphs by calculating successive powers of the associated adjacency matrix also called as Markov matrices which capture the mathematical concept of random walks on a graph [50].

### GO annotation analysis

To assess the identified candidate genes in the context of GO, the DAVID functional annotation tool (version 6.7) [38, 39] was used. The functional annotation clustering of significantly over-represented GO term: cellular compartment (CC), molecular function (MF) and biological process (BP) was retrieved by using options GOTERM_CC_ALL, GOTERM_MF_ALL and GOTERM_BP_ALL. The default setting parameters and multiple corrections by the Benjamini method were used to determine the significant enrichment score of 1.3 [38, 39].

### AD putative biochemical biomarkers analysis

To identify putative biochemical biomarker associated with AD, CSF and plasma proteins were retrieved from Sys-BodyFluid Database [146]. The 108 target genes were mapped to their corresponding uniprot ids using ID mapping tool available at http://www.uniprot.org[142]. The venn diagram of the overlapping proteins in all the three datasets (GWL,GWA and GWE) was created using GeneVenn tool [147] by taking intersection among these data sets.

## Electronic supplementary material

Additional file 1: **AD GWA and GWE replication datasets.** The file contains the list of 294 genes from AD GWA replication dataset (Boada et al.) and list of 182 genes from AD GWE replication dataset (Webster et al., GEO:GSE15222). (XLSX 56 KB)

Additional file 2: **Final list of AD genes from three data sets and final list of ranked 108 genes.** The file contains the list of genes from three datasets, final overlapping 108 genes ranked by their cumulative rank score. (XLSX 385 KB)

Additional file 3: **Venn diagrams of overlapping genes from independent analysis of genes from 6 brain region separately with GWA and GWL datasets.** The file contains Venn diagrams of genes from three datasets, final overlapping 108 genes ranked by their cumulative rank score. (PNG 2 MB)

Additional file 4: **List of overlapping genes from independent analysis of genes from 6 brain region separately with GWA and GWL datasets.** The file contains the list of genes from three datasets, final overlapping 108 genes ranked by their cumulative rank score. (XLSX 11 KB)

Additional file 5: **Clusters identified from PPI using MCL algorithm implemented in clusterMaker.** The file details the 69 clusters identified by MCL algorithm from the PPI containing 640 genes. (PDF 77 KB)

Additional file 6: **Annotation clusters from DAVID.** The file contains top 11 and 10 annotation clusters with GO analysis from DAVID for 108 and 640 genes respectively. (XLSX 22 KB)

Additional file 7: **Putative AD specific biomarkers among 640 AD proteins and proteins from CSF and plasma proteome.** The file contains Venn diagram showing overlap of 640 AD proteins and proteins from CSF and plasma proteome. (PNG 440 KB)

Additional file 8: **Final list of PD genes from three data sets and final list of 59 candidate overlapping genes.** The file contains the list of genes from three datasets, final overlapping 59 candidate genes. (XLSX 319 KB)

## Abbreviations

AD: Alzheimer’s disease
GWE: Genome Wide Expression
GWL: Genome Wide Linkage
GWA: Genome Wide Association
APP: Amyloid beta (A4) precursor protein
PSEN1: Presenilin 1
PSEN2: Presenilin 2
CDC2: Cyclin-dependent kinase 1
IRAK1: Interleukin-1 receptor-associated kinase 1
CSF: Cerebrospinal fluid
LOAD: Late onset Alzheimer’s disease
PPI: Protein-Protein Interaction
SNP: Single nucleotide polymorphism
MCL: Markov Clustering algorithm
GO: Gene Ontology.
