# Supplementary figures and images for: Genomic convergence and network analysis approach to identify candidate genes in Alzheimer's disease
Source: BMC Genomics. 2014 Mar 15;15(1):199. doi: 10.1186/1471-2164-15-199 (PMC4028079; doi:10.1186/1471-2164-15-199)

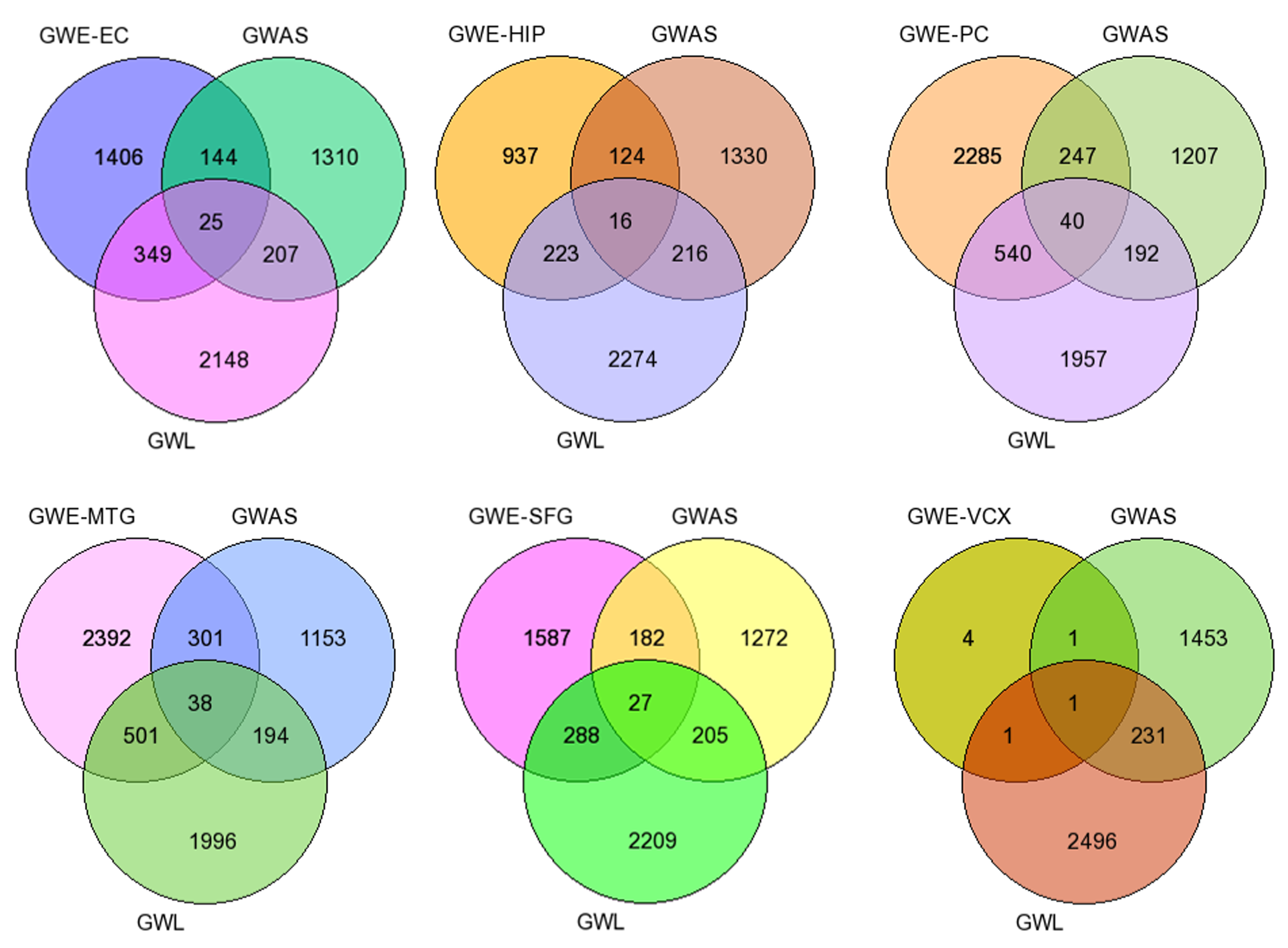

Supplement: Supplementary file 3 — Additional file 3: Venn diagrams of overlapping genes from independent analysis of genes from 6 brain region separately with GWA and GWL datasets. The file contains Venn diagrams of genes from three datasets, final overlapping 108 genes ranked by their cumulative rank score. (PNG 2 MB) [file 12864_2013_7037_MOESM3_ESM.png]

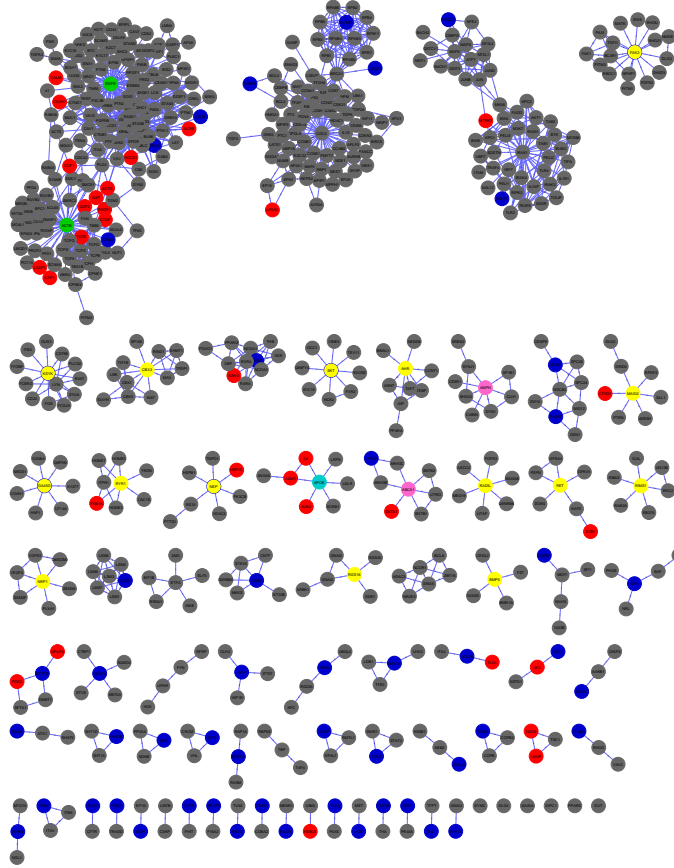

Supplement: Supplementary file 5 — Additional file 5: Clusters identified from PPI using MCL algorithm implemented in clusterMaker. The file details the 69 clusters identified by MCL algorithm from the PPI containing 640 genes. (PDF 77 KB) [file 12864_2013_7037_MOESM5_ESM.pdf]

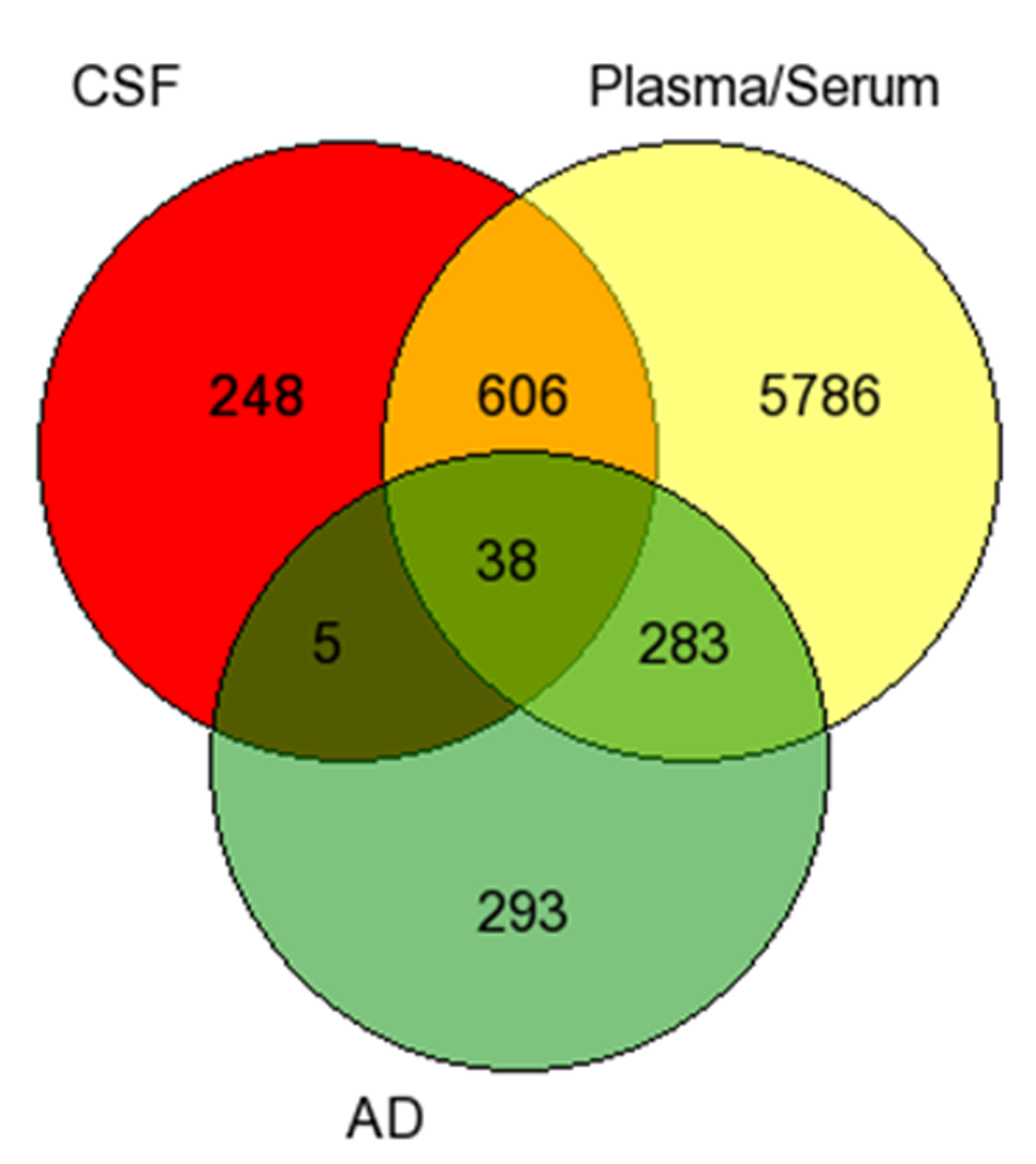

Supplement: Supplementary file 7 — Additional file 7: Putative AD specific biomarkers among 640 AD proteins and proteins from CSF and plasma proteome. The file contains Venn diagram showing overlap of 640 AD proteins and proteins from CSF and plasma proteome. (PNG 440 KB) [file 12864_2013_7037_MOESM7_ESM.png]
